# Lipid emulsion therapy in women with recurrent pregnancy loss and repeated implantation failure: The role of abnormal natural killer cell activity

**DOI:** 10.1111/jcmm.16257

**Published:** 2021-02-05

**Authors:** Paula Renata Bueno Campos Canella, Ricardo Barini, Patrícia de Oliveira Carvalho, Daniela Soares Razolli

**Affiliations:** ^1^ Laboratory of Multidisciplinary Research São Francisco University USF Bragança Paulista Brazil; ^2^ Department of Obstetrics and Gynecology Campinas University (UNICAMP) Campinas Brazil; ^3^ Postgraduate Program in Health Sciences São Francisco University Bragança Paulista Brazil

**Keywords:** lipid emulsions, NK cell activity, recurrent pregnancy loss, repeated implantation failure

## Abstract

Altered immune and/or inflammatory response plays an important role in cases of recurrent pregnancy loss (RPL) and repeated implantation failure (RIF). Exacerbation of the maternal immune response through increased NK cell activity and inflammatory cytokines can cause embryo rejection leading to abortion or embryo implantation failure. Immunosuppressors or immunomodulators can help or prevent this condition. Currently, lipid emulsion therapy (LET) has emerged as a treatment for RPL and RIF in women with abnormal NK cell activity, by decreasing the exacerbated immune response of the maternal uterus and providing a more receptive environment for the embryo. However, the mechanisms by which the intralipid acts to reduce NK cell activity are still unclear. In this review, we focus on the studies that conducted LET to treat patients with RPL and RIF with abnormal NK cell activity. We find that although some authors recommend LET as an effective intervention, more studies are necessary to confirm its effectiveness in restoring NK cell activity to normal levels and to comprehend the underlying mechanisms of the lipids action in ameliorating the maternal environment and improving the pregnancy rate.

## 1 BACKGROUND

Recurrent pregnancy loss (RPL) was first defined by the Royal College of Obstetricians and Gynecologists as three or more consecutive miscarriages before the twentieth week of pregnancy, excluding ectopic, molar and biochemical pregnancies. More recently, RPL was redefined as two or more spontaneous losses of clinical pregnancies before completing 22 weeks of gestation, affecting around 1%‐2% of women.^1^, ^2^

Some cases of RPL can benefit from assisted reproduction techniques, among them in vitro fertilization (IVF), an approach where fertilization is performed outside of the body and then the embryo is transferred to the uterus; even so, the in vitro transfer can be unsuccessful. Repeated implantation failure (RIF) is a failure to achieve a clinical pregnancy in women under 40 years old after three or more consecutive transfer cycles of at least four good‐quality embryos.^3^

Although the RIF aetiology is not completely established, variables such as maternal age, elevated BMI, immunological factors, sperm quality, uterine alterations and psychological conditions should be considered to direct treatment approaches. The implantation rate in women under IVF can vary from 25% to 40% depending on the embryo transfer protocol, and about 10% of patients under IVF are affected with RIF.^3^, ^4^ Studies conducted in recent decade have been suggesting that immunological abnormalities such as self‐recognition of an embryo or foetus could contribute to the implantation failure and thus explain the occurrence of RPL. The abnormal inflammatory response in RPL and RIF includes increased expression of pro‐inflammatory markers, human leucocyte antigens and circulating natural killer (NK) cells.^5^ Given that, several randomized clinical trials have assessed immune modulators as an approach to address the RPL and/or RIF conditions.^6^, ^7^

Other recent studies discuss the effectiveness in RPL treatment of some immunomodulatory agents such as paternal leucocyte immunization (PLI), intravenous immunoglobulin (IVIg), filgrastim and intralipid.^8^, ^9^, ^10^, ^11^ Among these, the lipid emulsion therapy (LET) has emerged as a possible new intervention therapy for women stricken by RPL and RIF. The cellular mechanisms by which intralipid acts are not completely understood, but some authors believe that the lipid emulsion restores the NK cells' abnormal activity to normal levels thereby improving embryo implantation.^6^, ^11^, ^12^

In this review, we included only studies using LET to treat patients with a history of RPL and/or RIF conditions as a result of increased NK cell activity. The Medical Subject Headings (MeSH) “NK cells”, “NK cell activity”, “natural killer”, “lipid emulsion therapy”, “intralipid”, “intralipid therapy”, “recurrent implantation failure” and “recurrent pregnancy loss” were used in different combinations for searching in the MEDLINE/PubMed electronic database. The period from 2008 to 2020 concentrates most of the publications matching both the selected MeSH and the scope of the review.

## 2 THE ROLE OF NATURAL KILLER CELLS IN RPL AND RIF

Natural killer (NK) cells are a type of cytotoxic lymphocyte involved in the early, innate, immune response against tumour cells and viral infections.^13^ NK cell activity is independent of prior activation and triggers the secretion of cytokines such as TNF‐alpha and INF‐gamma.^14^ NK cells are able to lyse virus‐infected cells and non‐expressing human leucocyte antigen (HLA) cells, inducing cell death through apoptosis.^15^ NK cells also undergo interaction with the human G‐leucocyte antigen HLA‐G gene, which is highly expressed in the trophoblast to prevent the activity of NK cells and the self‐recognition of foetal antigens by the maternal immune system suggesting that abnormal expression of the HLA‐G gene is involved in recurrent abortions.^16^

There are different types of NK cells classified according to their surface antigen expression markers that include CD16 and CD56. CD16 is responsible for the antibody‐dependent cytotoxic action, and CD56 can differentiate into two subpopulations with CD56^dim^ being the most cytotoxic one and CD56^bright^ the less cytotoxic one, producing pro‐inflammatory markers such as IFN‐gamma and TNF‐alpha.^17^ In recent years, studies have confirmed that abnormal expression of NK cells surface markers in peripheral blood, endometrial and uNK cells is involved in RPL and RIF, suggesting that NK cells activity is involved in the achievement and maintenance of pregnancy.^18^, ^19^, ^20^, ^21^, ^22^

NK cells in the peripheral blood of healthy individuals range from 5% to 29% depending on the gender, stress, ethnicity and age. Confirming RIF or RPL based on the levels of NK cells in patients' peripheral blood is a controversial issue. Although the analysis of uterine NK cells (uNK) instead of peripheral blood is more robust, this method requires an invasive procedure.^23^, ^24^ Although a positive correlation between NK cells from peripheral blood and uNK cells has been observed, some authors have shown non‐correlation suggesting that peripheral NK cells and uNK cells have completely different phenotypes and functions.^23^, ^25^, ^26^ Recently, it was shown that women with RIF presented an increased percentage of NK cells in their blood compared to the control group, evidence of a positive correlation between peripheral blood and endometrial CD56 cells, suggesting NK cell activity as a potential marker of RIF.^27^

A study performed by Mariee et. al. detected NK cells in the peripheral blood and directly from the endometrium. The authors observed a positive correlation between the number of uNK cells and interleukin 15 (IL‐15) in stromal cells suggesting that IL‐15 may play a role in the control of uNK endometrial function or cell proliferation.^28^ Using endometrial biopsy analysis, a study evaluated uNK abundance in the endometrium of women with idiopathic recurrent miscarriage (IRM) compared to fertile women and found that uNK was increased in IRM patients, suggesting a uNK role in the pathophysiology of recurrent miscarriage.^29^

Control of the immunological response, especially the regulation of NK cell cytotoxicity, is important to ensure embryo implantation success. The embryo triggers the implantation and invasion of trophoblasts that produce the preimplantation factor (PIF). It was shown that synthetic PIF is able to inhibit NK cell‐mediated cytotoxicity by reducing NK CD69 expression to comparable levels in intralipid and intravenous gamma‐Ig therapies for patients with RPL.^30^ Strengthening the hypothesis of NK cell activity involvement in RPL or implantation failure, one study found increased CD69 expression in different peripheral NK cell subtypes in women with unexplained RPL at least two months after the second consecutive miscarriage compared to a control group, suggesting peripheral NK as a marker of altered immune response.^31^

The unexplained aetiology of RPL and RIF increases the interest in seeking new targets and treatments for those conditions. More recently, LET has emerge as a potential intervention to prevent those conditions, especially when NK cells display increased activity in peripheral blood and/or the endometrium and uNK cells. The LET studies and their findings are described in detail in the next section.

## 3 LIPID EMULSION COMPOSITIONS AND APPLICATIONS

Lipid emulsions (LEs) are a mixture of fatty acids (FAs), including the essential linoleic and α‐linolenic unsaturated fatty acids, which are not produced by the organism. In 1920, Yamahakawa was the first to administer intravenous LEs to humans, and in 1945, McKibbin et al established the use of lipid emulsion for parenteral nutrition. The LEs solutions only became commercially available in the 1950s.^32^ Currently, the commercial lipid emulsions are constituted of n‐3, n‐6 and n‐9 long‐chain triglycerides isolated or in association with medium chain triglycerides.^33^

Since the 1960s, LEs have excelled in parenteral nutrition therapy. The first generation of LEs was composed exclusively of soy oil (SO), containing a high percentage of n‐6 polyunsaturated fatty acids. In the 1980s, the second generation of LEs was elaborated with a lower percentage of n‐6 fatty acids compared to the first one. This second generation was composed of 50% coconut oil (CO) which is rich in medium chain triglycerides (MCT). In the 1990s, olive oil (OO) was introduced to LEs, giving rise to the third generation. Currently studies have shown the importance of MUFA/PUFA, and from the 2000s onwards, the n‐3 fatty acids family have been included in LEs, represented by the addition of fish oil (FO) ensuring the desired n‐6:n‐3 proportion.^34^

Isolated or mixed FO emulsion is a source of n‐3 and has anti‐inflammatory properties.^35^ Bae et al published a meta‐analysis showing reduced mortality and hospital stay in surgical patients receiving LEs with fish oil compared to patients receiving LEs without fish oil.^36^ Another study evaluated the effects of LEs from the n‐3 fatty acids family in septic patients and observed a reduction in arachidonic acid (AA) compared to the amount of EPA + DHA and that was associated with improved survival in those patients.^37^

In view of its anti‐inflammatory effect, LET has emerged as a potential candidate to ameliorate the RPL or RIF conditions in women. Among the commercially available LEs, the most used is Intralipid^®^; it consists mainly of purified soya bean oil (10% or 20%) and egg yolk phospholipids (2.25%) emulsified with glycerine and water. Table 1 shows all the commercially available LEs.

**TABLE 1:** Composition of commercially available lipid emulsions

Commercial name	Lipid source	Linoleic (%)	α‐Linolenic (%)	α‐Tocopherol, mg/L	Phytosterols, mg/L	ω‐6: ω‐3 ratio
Intralipid^®^ 10%, 20%, 30%	100% soya bean oil	44‐62	4‐11	38	348 ± 33	7:1
Structolipid^®^ 20%	64% soya bean oil 36% MCT	35	5	6.9	NA	7:1
Lipofundin^®^ MCT/LCT 10%, 20%	50% soya bean oil 50% MCT oil	27	4	85 ± 20	NA	7:1
ClinOleic^®^ 20%	20% soya bean oil 80% olive oil	18.5	2	32	327 ± 8	9:1
SMOFlipid^®^ 20%	30% soya bean oil, 30% MCT, 25% olive oil, 15% fish oil	21.4	2.5	200	47.6	2.5:1
Omegaven^®^ 10%	100% fish oil	4.4	1.8	150‐296	0	1:8

Note

Description and composition of the main lipid emulsions. Data supplied by the manufacturers.

Abbreviations: LCT, long‐chain triglyceride; MCT, medium‐chain triglyceride; NA, not available; ω‐6, ω‐3 Ratio, ratio of ω‐6 fatty acids to ω‐3 fatty acids.

## 4 LIPID EMULSION THERAPY IN RPL AND RIF

Taking into account the increased activity of NK cells reported in patients with RPL and RIF, and considering the possible anti‐inflammatory action of the polyunsaturated fatty acids from the lipid emulsions, the intralipid infusion is emerging as a possible therapy. For this review, we selected studies published from 2008 to 2020, where LEs were used as an intervention to treat RPL and RIF in women with abnormal NK cell activity.

From 2008 to 2020, five studies meeting the abovementioned criteria were published. Among them, three studies observed a significant decrease of NK cell activity when LET was administered, and two did not report any significant effect. Although the studies mention that women with high NK cell activity were chosen for the intervention, some authors fail to present complete data for the NK cell activity before and/or after treatment. Here, we describe in detail the main findings of the studies summarized in Table 2.

**TABLE 2:** Summary of the analysed studies

Authors and year of publication	Aim	Participants and Methods	Results
Roussev et al,^12^ 2008	To establish the duration and efficacy of Intralipid^®^ 20% infusion treatment in suppressing NK cell activity in patients with reproductive failure	50 women with abnormal NK cell activity received three Intralipid^®^ 20% infusions.	In the third Intralipid^®^ infusion, all participants showed normal NK cell activity. The suppressive effect lasted mostly 6‐9 weeks.
Dakhly et al,^6^ 2016	To determine the efficacy of Intralipid^®^ 20% infusion in women with recurrent spontaneous abortion and abnormal NK cell activity submitted to in vitro fertilization (IVF)/ intracytoplasmic sperm injection (ICSI) cycles	296 women with increased NK cell activity participated in a randomized, double‐blind, controlled trial, of which: n = 144 received three Intralipid^®^ 20% infusions and n = 152 received placebo	Chemical pregnancy was achieved in 58.3% of women from Intralipid^®^ group and 50.0% of women from the control group, suggesting that Intralipid^®^ infusions did not increase the frequency of chemical pregnancy
Meng et al,^7^ 2016	To determine whether intralipid can be used as an alternative treatment to the intravenous immunoglobulin (IVIG) treatment in women with abnormal natural killer cell activity	154 women distributed in 2 groups, of which: n = 76 received intralipid infusion and n = 78 received intravenous IVIG infusion. Both intralipid and IVIG were infused at different times.	There was no statistically significant difference in successful pregnancy rates between the two groups (92.1% versus 88.2%, *P* = .415).
Lédée et al,^19^ 2018	To investigate whether Intralipid^®^ 20% therapy has NK cell immunosuppressive properties	Total of 94 patients with a history of RIF and NK cell over‐immune activation between 2012 and 2017.	The intralipid showed a 54% of live birth rate in women after embryo transfer. Also, a reduction of the over‐immune endometrial markers (CD56; IL‐18/TWEAK; IL‐14/FN‐14) was observed
Martini et al,^5^ 2018	To determine whether intralipid infusion improves live birth rate in RPL and RIF women with elevated peripheral NK cells activity and to confirm whether intralipid is a cost‐effective therapy.	A retrospective cohort study was performed with 127 patients who underwent intralipid therapy from 2012 to 2015 compared to n = 20 from historical cohort data.	Intralipid infusion did not improve live birth rates and was more expensive compared to control group in patients with RIF or RPL.

Note

Description of the studies included in this review, according to the authorship and year of publication, aim, participants included, methods performed and the main findings.

Roussev et al (2008), Meng et al (2016) and Lédée et al (2018) observed a decrease in NK cell activity and share the idea that LET is an option to treat RPL and RIF conditions. In the opposite direction, Dakhly et al (2016) observed an increase in the rate of ongoing pregnancies and live births; however, there was no increase in the chemical pregnancy rate. In addition, the authors mentioned that women with abnormal NK cell activity were included in the study, but the data for NK cell activity were not shown. Martini et al (2018) did not observe any increase in the live birth rate in a retrospective intralipid cohort (n = 127) study compared with the historical control cohort (n = 20) data.^5^, ^6^, ^7^, ^12^, ^19^

Roussev et al analysed fifty patients with abnormal NK cell activity. The patients selected for 20% intralipid solution infusion had NK cell activity checked weekly. The authors showed that 78% of patients presented suppression of NK cell activity in the first week after infusion. However, for 22% of the patients, second and third infusions were necessary to attain normal levels of NK cell activity. In 47 patients, the suppressive effect of intralipid in NK cells lasted 6‐9 weeks, and in 3 patients, the suppression lasted 4‐5 weeks. The authors stated that the main advantages of LET are the low cost and long‐lasting effects when compared to other therapies. They suggested that the fatty acids present in the lipid emulsions can act as ligands to activate peroxisome proliferator‐activated receptors (PPARs) expressed by NK cells, reducing their activity. Activation of PPARs has been shown to decrease the cytotoxic activity of NK cells, so it is possible that the intralipid modulates the immune system favoring embryo implantation and pregnancy maintenance.^12^

Meng et al conducted a prospective, randomized, clinical trial between December 2010 and December 2012 and investigated whether intralipid is an immunosuppressive treatment as effective as intravenous immunoglobulin (IVIG), which is expensive and has many side effects. The participants were divided into an intralipid group and an IVIG group. The first intralipid 20% infusion in 250 mL of saline was administered on the third day of the menstrual cycle for at least 2 hours. Thereafter, infusions were administered every 2 weeks before and once a week after pregnancy until the 12th week of pregnancy. The primary outcome was the successful pregnancy rate. In addition, percentage comparisons of peripheral NK cell activity were performed by flow cytometry and were compared before and after each treatment. The results obtained showed non‐significant differences in successful pregnancy rates between intralipid and IVIG (92.1% vs 88.2%, *P* = .415). The decreased NK cell concentrations revealed the cytotoxic effects of treatments in both groups; the authors affirm that LET can be used with the same effectiveness as IVIG to treat patients with elevated NK cell activity.^7^

Lédée et al carried out a randomized control trial between 2012 and 2017 where 94 patients with a history of unexplained RIF and endometrial over‐immune activation received a slow infusion of LE (Intralipid^®^) before embryo transfer. The analysis of NK cells in this study was through endometrial biopsy performed by aspiration with a Cormier pipette in the middle luteal phase. The gene expressions of IL‐15/Fn14 and IL‐18/TWEAK were determined by quantitative RT‐PCR, and uNK CD56 + positive cells were verified by immunohistochemistry. An association of three biomarkers was used to define the uterine immune profile. The proportion of IL‐18/TWEAK reflects the locally immuno‐regulated Th1/Th2 balance and local angiogenesis; the proportion of IL‐15/Fn‐14 reflects the maturation of uNK cells and the number of CD56 positive cells. The activated immune profile was characterized by a high ratio of IL‐18/TWEAK and/or a high proportion of IL15/Fn‐14.^19^

The infusion was administered around day 8 of the embryo transfer cycle (100 mL Intralipid^®^ 20% in 400 mL of saline for 90 minutes). When pregnancy was confirmed, new infusions were performed on the fifth and ninth weeks of amenorrhoea. Among the 94 patients with over‐immune activation, 60% had a local excess of Th‐1 cytokines (high IL‐18/TWEAK ratio); 57% showed uNK cell over activation via IL‐15 (high proportion of IL‐15/FN‐14); and 37% had an excessive recruitment of CD56. In patients who received intralipid emulsion, the authors found a significant decrease in the three biomarkers used to confirm over‐immune endometrial activation. The decrease in IL‐18/ TWEAK is mainly induced by the decrease in pro‐inflammatory cytokine IL‐18. The significant decrease in IL‐15/FN‐14 is mainly caused by a decrease in IL‐15 expression. Intralipid^®^ appears to decrease the over activation of uterine NK cells through regulation of the recruitment and expression of the pro‐inflammatory cytokines.^19^

Dakhly et al performed a double‐blind, randomized, controlled trial at Cairo University from February 2013 to April 2015. The study included women with unexplained infertility and increased NK cell activity (>12%) undergoing in vitro fertilization/intracytoplasmic sperm injection. The women were divided in n = 144 for the intralipid group and n = 152 for the control group. The intralipid group received 2 mL of intralipid infusion 20% in 250 mL of saline on the day of the embryo transfer or insemination. After positive pregnancy, the intralipid infusion was repeated every 2 weeks until the end of the first trimester. The authors observed a significant frequency of ongoing pregnancies and live births rate (*P* value of .005 for both) in the intralipid group; however, in chemical pregnancy, the effect was not observed. The study mentions that the women presented an increase in NK cell activity, but they did not include or mention the number, percentage or expression of NK cells.^6^

Martini et al performed a retrospective cohort study at a large private infertility clinic from 2012 to 2015. For the study, they selected 127 patients with increased peripheral NK cell activity and a history of RPL and RIF and they received Intralipid. The analyses of NK cell activity were performed by flow cytometry at different time‐points, the first at least 2 weeks before the intervention and then repeated weekly. The authors considered that values above 19% were high for NK activity (aNK), and the intralipid infusion aimed to reach aNK below 10%. Over a period of 90 to 120 minutes, 4 mL of intralipid infusion 20% plus 250 mL 0.9% saline solution was administered 7‐10 days before embryo transfer or insemination. After pregnancy was achieved, the infusion was repeated at approximately 6 and 10 weeks of gestation. The authors are against LET as a treatment for RPL; however, they recommend that research should focus on the standardization and the development of a secure method to confirm the NK cell activity, as currently there is no standard for analysis. Although the authors chose patients with increased NK cell activity and history of RPL and RIF for the study, they do not show or comment on whether there was any decrease in NK cell activity in the treated patients.^5^

Lipid emulsion therapy has been proposed as a valid and promising alternative for the treatment of RPL and RIF in women with abnormal NK cells activity. When compared to IVIG therapy, intralipid infusion did not show any significant difference in the rate of live births in women with a history of embryo implant failure, recurrent abortion and high NK cell cytotoxicity.^7^, ^38^ It has been shown that 200 patients with RPL and RIF with increased NK cell activity had 61% of live births after LET, which did not differ significantly from the 52% observed for intravenous therapy with immunoglobulin.^38^ In other words, LET has been shown to be as effective as immunoglobulin but with the advantage of not being derived from blood and having a lower cost.^38^ Nevertheless, such findings should be considered with caution, as more studies are necessary to confirm the results and explain the mechanisms by which lipid emulsions suppress NK cell activity in RPL and RIF.^12^, ^39^

Although the mechanism by which intralipids regulate NK cells function is still unclear, the fatty acids present in intralipid can be recognized by peroxisome proliferator‐activated receptors (PPARs), G‐protein‐coupled receptors (GPCR) and cluster of differentiation (CD1) receptors. Once intralipid particles enter NK cells, they activate signalling pathways involved in immune response, fatty acids activation and transportation. Furthermore, intralipids have been shown to stimulate the reticuloendothelial system to remove ‘danger signals’ that can lead to pregnancy loss.^11^

According to the reviewed papers, the infusion protocols and results obtained with LET are not consensual and more studies are necessary to confirm its efficacy in improving live birth rates. The beginning of intralipid treatment can vary from the day of oocyte retrieval, the third day of menstrual cycle, 7‐10 days before embryo transfer or insemination to day 8 of the embryo transfer. Most authors used around 3‐12 infusions of a 20% LEs administered over 30‐120 minutes diluted in saline to reach the aimed concentration and guarantee the slow infusions recommended for LEs. Before pregnancy, the intralipid infusion is given every 2 weeks and, once pregnancy is confirmed, the infusion protocol differs among the authors from once a week to every 2‐4 weeks. Most of the authors end the treatment by the 12th week of gestation. Moreover, large‐scale studies, double‐blind placebo‐controlled trials need to be performed in different populations to test the efficacy of LET before it can be recommended for routine use.^19^, ^40^, ^41^

To sum up, sixty per cent of the reviewed studies obtained satisfactory results demonstrating that LET contributed to decreasing NK cell activity in patients with RPL and RIF. Twenty per cent mentioned that they achieved satisfactory and significant results with the LET, although the data for NK cell activity were not addressed in the results and discussion, that would have been interesting to confirm whether there had been a significant reduction of those cells. Twenty per cent did not obtain satisfactory results with LET and mentioned that studies are necessary to define secure protocols for the analysis of NK cell activity, whether peripheral or endometrial, as well as the protocol for intralipid dilution and infusion.

## 5 CONCLUSION

Currently, RPL and RIF caused by inflammatory or immunological abnormalities are increasingly common. Although there are only a few studies published on the field, lipid emulsion therapy has been proposed as an immuno‐suppressor of the activity of NK cells and other inflammatory biomarkers which could contribute to a viable pregnancy in patients with a history of RPL and RIF. Some studies have observed an increase in implantation and live births and a decrease in the activity of NK cells after intralipid infusions. However, more studies are necessary to verify the mechanism by which LEs acts to decrease the activity of NK cells. Another important factor that must be considered is the composition of the lipid emulsions, as the n‐6:n‐3 ratio is essential to promoting increase or reduction in the immune inflammatory response. In conclusion, the LEs are a promising option to treat patients with RPL and RIF but studies focusing on NK cell activity must be performed in order to understand the LEs mechanism in RPL and RIF and promote a better comprehension of the pathophysiology of these conditions.

## CONFLICT OF INTEREST

The authors have declared no conflict of interest.

## AUTHOR CONTRIBUTIONS

**Paula Renata Bueno Campos Canella:** Conceptualization (lead); writing‐original draft (lead); writing‐review and editing (lead). **Ricardo Barini:** Writing‐original draft (supporting). **Patricia de Oliveira Carvalho:** Conceptualization (lead); writing‐original draft (supporting); writing‐review and editing (supporting). **Daniela Soares Razolli:** Conceptualization (lead); writing‐original draft (lead); writing‐review and editing (lead).

## Data Availability

Data sharing is not applicable to this article as no new data were created or analysed in this study.
